# Do Health Claims and Front-of-Pack Labels Lead to a Positivity Bias in Unhealthy Foods?

**DOI:** 10.3390/nu8120787

**Published:** 2016-12-02

**Authors:** Zenobia Talati, Simone Pettigrew, Helen Dixon, Bruce Neal, Kylie Ball, Clare Hughes

**Affiliations:** 1 School of Psychology and Speech Pathology, Curtin University, Kent St, Bentley, WA 6102, Australia; simone.pettigrew@curtin.edu.au; 2 Centre for Behavioural Research in Cancer, Cancer Council Victoria, Melbourne, VIC 3004, Australia; helen.dixon@cancervic.org.au; 3 The George Institute for Global Health, University of Sydney, Sydney, NSW 2000, Australia; bneal@georgeinstitute.org.au; 4 Institute for Physical Activity and Nutrition, School of Exercise and Nutrition Sciences, Deakin University, Geelong, VIC 3220, Australia; kylie.ball@deakin.edu.au; 5 Cancer Council, Sydney, NSW 2150, Australia; clareh@nswcc.org.au

**Keywords:** positivity bias, health halo, front-of-pack labelling, daily intake, traffic lights, health star rating, health claims

## Abstract

Health claims and front-of-pack labels (FoPLs) may lead consumers to hold more positive attitudes and show a greater willingness to buy food products, regardless of their actual healthiness. A potential negative consequence of this positivity bias is the increased consumption of unhealthy foods. This study investigated whether a positivity bias would occur in unhealthy variations of four products (cookies, corn flakes, pizzas and yoghurts) that featured different health claim conditions (no claim, nutrient claim, general level health claim, and higher level health claim) and FoPL conditions (no FoPL, the Daily Intake Guide (DIG), Multiple Traffic Lights (MTL), and the Health Star Rating (HSR)). Positivity bias was assessed via measures of perceived healthiness, global evaluations (incorporating taste, quality, convenience, etc.) and willingness to buy. On the whole, health claims did not produce a positivity bias, while FoPLs did, with the DIG being the most likely to elicit this bias. The HSR most frequently led to lower ratings of unhealthy foods than the DIG and MTL, suggesting that this FoPL has the lowest risk of creating an inaccurate positivity bias in unhealthy foods.

## 1. Introduction

Nutritional information on the front of food packs (i.e., health claims and front-of-pack labels (FoPLs)) can help inform consumers about the health value of food products. However, through its mere presence, such information has been found to positively bias consumers’ perceptions of products [1,2]. A positivity bias occurs when consumers evaluate products more favourably as a result of the presence of on-pack nutrition information (e.g., health claims or FoPLs), compared to similar products that do not display this information, regardless of the healthiness of the product’s nutrient profile [3]. This bias can therefore lead consumers to incorrectly judge an unhealthy product as healthier than they would if no on-pack nutrition information was present, which has the potential to encourage consumers to increase their consumption of these foods if they overestimate their nutritional value [4,5].

The positivity bias differs somewhat from the halo effect, another cognitive bias in which consumers generalize specific nutrient information to other product attributes (e.g., assuming a product low in cholesterol is also low in fat [3]). A positivity bias towards foods with health claims may be explained by the fact that health claims only provide positive information relating to selected nutrients and are generally easier to understand than the Nutrition Facts Panel [5,6]. FoPLs, however, provide a balanced summary of overall product healthiness and often convey negative information. Thus the underlying mechanism of the positivity bias in FoPLs is less clear.

## 2. Health Claims

Although foods with health claims tend to be marginally healthier than similar products without a claim, such claims frequently appear on products that would be classified as unhealthy [7,8]. Furthermore, the frequency with which different diseases are mentioned in health claims does not reflect the real world burden of these diseases (i.e., diseases with greater Disability Adjusted Years of Life lost are mentioned less frequently in health claims) [9]. Health claims can therefore potentially create a positivity bias through the provision of one-sided, positive nutrition information [10,11,12,13,14,15]. It is not uncommon for consumers to make global inferences about a product based on specific information (such as that contained in health claims) and subsequently seek out information to support this perspective [16,17].

There is evidence a positivity bias can be induced through various types of health claims, including nutrient claims (which describe the presence, absence or level of nutrients within a product or compared to other products), general level health claims (which link nutrients in the food to a positive physical outcome) and higher level health claims (which describe specific diseases affected by nutrient consumption) [3,18,19,20,21,22,23,24,25,26,27,28]. However, there is limited information on whether this effect applies equally to healthy and unhealthy foods since most studies either do not manipulate healthiness, use a choice-based design in which participants select the healthiest option (thus providing no information on their judgements of the unhealthy option) [29], or do not test for an interaction between healthiness and the presence or absence of a health claim [6].

Of those studies that have assessed the positivity bias of nutrient claims in unhealthy foods, the findings are mixed [4,18,29,30,31,32,33]. Some studies have reported no difference between claim and no claim conditions in terms of perceived healthiness, overall attitude to the product and purchase intentions [30,31]. However, in the presence of a claim other studies have reported a positivity bias in terms of willingness to pay [18], increased consumption, increased perceptions of appropriate serving size and decreased calorie estimation [4] while others reported mixed findings across multiple outcomes [29,32,33]. As an example of the latter, Dixon and colleagues found that nutrient claims on unhealthy products led participants to perceive that target nutrients were present in higher quantities and made them more likely to select the product from a choice set including similar but healthier products. However, no significant differences were found in overall ratings of product healthiness, taste or purchase intentions [29,32].

The research on general and higher level claims is even more limited than that for nutrient claims. Of the three studies that could be identified examining unhealthy food products, one found evidence of more positive evaluations resulting from general level health claims [31] while others found that a higher level claim on unhealthy products led to increased perceptions of healthiness and willingness to try [34], a greater willingness to pay [18] or mixed results across different outcome variables [35]. Altogether, research to date suggests that certain types of claims may lead to a positivity bias for unhealthy foods. This led to the following hypothesis:

*H1*: General and higher level health claims will lead to a positivity bias (i.e., increased perceptions of healthiness, global evaluations and/or willingness to buy compared to no health claim) in unhealthy foods, while nutrient claims will not.

## 3. Front-of-Pack Labels

**Figure 1:**
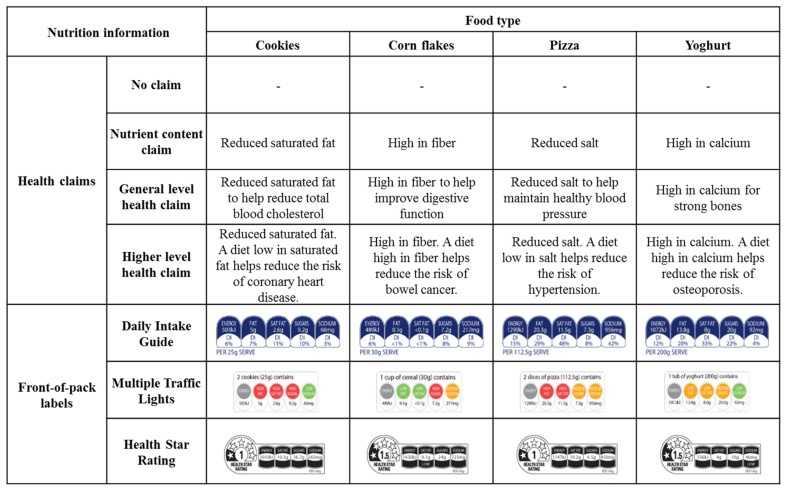
Health claims and front-of pack labels by food type.

In contrast to health claims, FoPLs are considered to provide more of a comprehensive, balanced summary of a food’s healthiness [36]. Multiple FoPLs can and often do exist in a marketplace. In Australia, where the present study was conducted, the Daily Intake Guide and Health Star Rating FoPLs are both used in supermarkets and the Multiple Traffic Light FoPL is used to determine the suitability of foods to be sold in many school canteens, hospitals and work places (see Figure 1 for examples of these FoPLs). FoPLs can be classified on a scale from reductive (i.e., FoPLs that contain reduced nutritional facts only) to evaluative (i.e., FoPLs that provide some form of recommendation about product healthiness) [2]. Purely reductive FoPLs such as the Daily Intake Guide have been criticized for being too complex and requiring greater cognitive load to interpret [37,38], while purely evaluative FoPLs such as logos may be considered oversimplified [36,39]. The Multiple Traffic Light and Health Star Rating system are hybrid FoPLs since the reductive information (i.e., nutrients in grams) is overlaid with an evaluative component (i.e., colours in the Multiple Traffic Light and the option to classify nutrients as “low” and “high” in the Health Star Rating). The main difference between these two hybrid systems is that through its overall star rating the Health Star Rating provides an evaluation of the product as a whole, whereas the Multiple Traffic Light does not.

Much like the health claims literature, the designs and methods of analysis of many FoPL studies preclude an understanding of whether a positivity bias occurs for unhealthy foods. One commonly adopted design involves presenting participants with choice sets containing healthy and unhealthy products that either do or do not feature a FoPL and measuring how frequently the healthiest option is chosen. In these studies, FoPLs with an evaluative component (i.e., the Multiple Traffic Light) generally demonstrate superior performance compared to reductive FoPLs (i.e., the Daily Intake Guide) [40,41,42,43]. However, the use of FoPLs to increase uptake of healthy foods is only desirable if the same outcome does not also occur in unhealthy foods. Designs that are restricted to food choice tasks between healthy and unhealthy products, do not allow assessment of how unhealthy foods are perceived and how consumers would react to choice sets where there is no healthy option (i.e., would participants choose an unhealthy food product or choose to opt out?) or where some products do not contain a FoPL (since not all products in a marketplace contain FoPLs).

The other commonly adopted design in the literature involves participants rating products that vary by FoPL to assess liking of FoPLs, attention to FoPLs, perceived product healthiness and/or understanding of nutrition information. Some of these studies do not manipulate healthiness or include a no FoPL control condition. In those that do, the effect of FoPLs on unhealthy foods is often not calculated or reported (generally because healthiness is not manipulated) or the data analysis incorporates other variables that prevent estimation of this interaction [40].

Nine studies could be found that analysed the isolated effect of FoPLs on judgements of unhealthy foods [1,2,31,43,44,45,46,47,48]. Most studies comparing the Daily Intake Guide to a no FoPL condition found that participants were more likely to: (i) perceive a product with a Daily Intake Guide as healthier and more favourable overall; and (ii) express greater willingness to buy, regardless of healthiness [2,31,47]. Only one study reported that the Daily Intake Guide reduced perceptions of healthiness compared to no FoPL [45]. Most studies on the Multiple Traffic Light reported a significant decrease in perceived healthiness and purchase intentions for unhealthy foods when the Multiple Traffic Light was present compared to no FoPL or the Daily Intake Guide [31,43,44,45,46,48]. Only one study found a positivity bias in unhealthy products featuring a Multiple Traffic Light [2].

With its very recent entrance into the Australian and New Zealand marketplaces, far less research has been conducted on the Health Star Rating. In the one study that could be located, participants were less likely to choose a product, regardless of healthiness, if the Health Star Rating was present on-pack [1]. Of note is that this study was conducted before the Health Star Rating was implemented in supermarkets and the five-star graphic used on the food stimuli in this study was coloured red [1]. When used in the context of food, the colour red can implicitly evoke an avoidance reaction, regardless of actual healthiness [49]. Similarly, research on the Multiple Traffic Light indicates that consumers are strongly driven to avoid red lights (more so than to select green lights) [50,51,52]. This negative colour association combined with a lack of familiarity with the Health Star Rating could have contributed to participants forming an overall negative impression of all products (healthy and unhealthy) with a Health Star Rating in that study [1].

In summary, the limited research to date suggests that the Daily Intake Guide is likely to induce a positivity bias when present on packs while the Multiple Traffic Light may help consumers more accurately judge the healthiness of unhealthy foods. Since the Health Star Rating and Multiple Traffic Light both contain interpretive information, it was theorized that these two FoPLs would produce similar outcomes. This led to the following hypothesis:

*H2*: The Daily Intake Guide (a reductive FoPL) will lead to a positivity bias in unhealthy food (i.e., increased perceptions of healthiness, global evaluations and/or willingness to buy) while the Multiple Traffic Light and Health Star Rating (hybrid FoPLs) will not.

In addition to the above hypotheses, this study explored whether unhealthy variations of different types of foods (i.e., cookies, corn flakes, pizzas and yoghurts) were more or less likely to exhibit a positivity bias in the presence of varying forms of nutrition information. A range of product categories was selected for inclusion in the study to accommodate the fact that consumer judgments of product healthiness are dependent on both the product category and the type of benefit claimed [53,54]. For example, health claims are generally seen as more acceptable on products that are already considered healthy [55,56]. For products that are seen as more hedonic, emphasizing health benefits can have a negative impact on taste evaluations [57,58]. Health claims and FoPLs were both included in this study because the coexistence of these two forms of nutrition information reflects common practice in many national marketplaces. The limited research looking at consumers’ responses to products featuring both forms of nutrition information suggests that they may prioritize the information presented in FoPLs over health claims [31,59,60]. The interaction between these two forms of nutrition information was therefore explored in this study. As per previous studies, the positivity bias was operationalized along the three dimensions of perceived healthiness, global evaluations and willingness to buy [1,44,47,61].

## 4. Methods

This study was part of a larger research project investigating Australian consumers’ attitudes towards packaged foods (Trial ID: ACTRN12616000626460, see https://www.anzctr.org.au/Trial/Registration/TrialReview.aspx?id=370675 [62] for further details on all of the variables manipulated and assessed). Ethics clearance was obtained from the Curtin University Human Research Ethics Committee (Approval number: RDHS-11-15, date of approval: 15 January 2015). The study design and variables relevant to the present analysis are described below.

### 4.1. Participants

Australian adults and children (aged 10 years and over) were invited to participate in a national online survey, which was hosted by an ISO accredited web panel provider (PureProfile). A total of 2058 participants took part in the larger project, with data from 1984 participants relevant to the present study. Quotas were set in regards to age, gender and socioeconomic status (SES) [63] to ensure equal representation of both genders and adequate representation of understudied groups such as children (23% of sample) and people from low SES neighbourhoods (48% of sample) [64,65]. Table 1 shows the characteristics of the final sample.

### 4.2. Procedure

Consent was obtained from all adults and children participating in the study, with parental consent also obtained for the children. Participants were asked about the frequency with which they purchase and consume the foods shown in the survey (i.e., cookies, corn flakes, pizzas, yoghurts). Those who indicated that they “never” purchase or consume more than 2 of the 4 foods used in the survey were deemed ineligible. Each participant was then shown 8 mock packs (one at a time) and had the option to click to view the NIP. Mock packs were presented randomly with the following constraints: (a) the first 2 mock packs came from the no FoPL condition; (b) for the remaining 6 mock packs, 2 mock packs came from each of the 3 FoPL conditions; and (c) across all 8 mock packs, 2 came from each of the 4 food type conditions. This resulted in a within and between subjects design.

Participants rated each mock pack on its perceived healthiness, global evaluation and willingness to buy. Perceived healthiness was assessed by taking the average of two 5-point adjective rating scales: unhealthy–healthy and non-nutritious–nutritious (Cronbach’s α = 0.91). Global evaluation was assessed by taking the average of nine 5-point adjective rating scales: Not tasty at all–Tasty, Low quality–High quality, Poor value for money–Good value for money, Inconvenient–Convenient, Boring–Interesting, Unpopular–Popular, Expensive–Cheap, Undesirable–Desirable, and Unappealing–Appealing (Cronbach’s α = 0.90). Willingness to buy was measured on a 5-point scale using the item: “Assuming you were interested in purchasing this type of food, how likely would you be to buy this specific item?”.

### 4.3. Stimuli

Mock packs were created by a graphic designer to resemble packaging currently found in Australian supermarkets. The mock packs relevant to this study varied by food type (cookies, corn flakes, pizza, yoghurt), FoPL (none, Daily Intake Guide (DIG), Multiple Traffic Light (MTL), Health Star Rating (HSR)) and health claim (none, nutrient content, general level, higher level). Each cell in this 4 × 4 × 4 design contained on average 85 observations (range: 79–92). Since this project was concerned with examining potential detrimental effects of a positivity bias when evaluating unhealthy products, only the mock packs that were designed to be unhealthy (i.e., with a star rating or equivalent of 1 or 1.5) were included in the analysis. The nutritional profile of each product was based on existing similar products in the Australian marketplace. The specific health claims and FoPLs used in the current study are shown in Figure 1. Of the FoPLs used in this study, respondents would have been most familiar with the DIG (which has been used in Australian supermarkets for a decade) and less familiar with the MTL (which has been used in school canteens in recent years and appears on some pre-packaged children’s foods) and the HSR (which, at the time of the survey, had begun to appear in supermarkets in the previous year).

### 4.4. Analysis

Mixed effects linear models were run on ratings of perceived healthiness, global evaluations and willingness to buy across the pooled data for all four food types. This analytical approach is recommended for use in studies where each participant provides multiple observations [66]. Health claim condition, FoPL condition and the interaction between the two variables were entered as fixed effects. Age, gender and SES were entered as covariates and participant identifier was entered as a random effect. Planned comparisons (with a Sidak adjustment for multiple comparisons) were then conducted to determine where the differences occurred between the health claim and FoPL conditions. The same analyses and planned comparisons were conducted on the data after it was split according to food type. The criterion for significance was set at *p* ≤ 0.05.

## 5. Results

The results from the pooled analyses (with data from all food types) are reported first, with a description of main effects and planned comparisons (which indicate the nature and direction of these effects). This is followed by analyses by specific food type to demonstrate how the effects varied according to food product category. When reporting these results, a positivity bias was considered to occur when the presence of a FoPL or a health claim led to more positive ratings compared to the control condition on at least one of the three outcome measures of perceived healthiness, global evaluations and willingness to buy.

### 5.1. All Foods (Pooled Data)

Table 2 presents the means, main effects and interaction effects of the linear mixed models across all outcomes for the data pooled across all foods. FoPLs were significant predictors across all outcome variables while health claims and the interaction between health claims and FoPLs were not.

**Figure 2:**
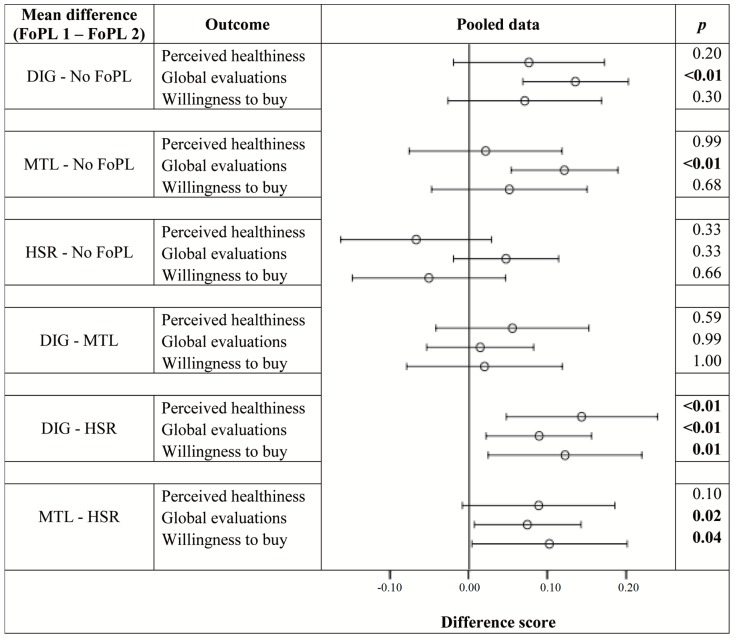
Difference in ratings between FoPLs (⊖) and the upper (├) and lower (┤) bounds of 95% confidence intervals for perceived healthiness, global evaluations and willingness to buy for pooled data.

Figure 2 graphically presents the results of the planned comparisons between FoPL conditions across all outcomes. As can be seen, the DIG and MTL led to a positivity bias in global evaluations (i.e., there was a positive and significant difference when ratings from the no FoPL condition were subtracted from ratings of foods with these FoPLs). Although no other positivity biases were observed, the DIG consistently led to higher ratings than the HSR across all outcomes. The HSR did not differ from no FoPL (control condition) for any outcome variables.

### 5.2. Analysis by Food Type

Table 3 presents the means, main effects and interaction effects for the linear mixed models across all outcomes by food type. Health claims were a significant predictor in only one instance, with higher level health claims producing a positivity bias for willingness to buy cookies. Since there were no other significant differences between health claims, planned comparisons for each food type were not carried out. FoPLs, however, were a significant predictor of at least one of the three outcomes in each food type, and hence planned comparisons were conducted across all outcomes.

**Figure 3:**
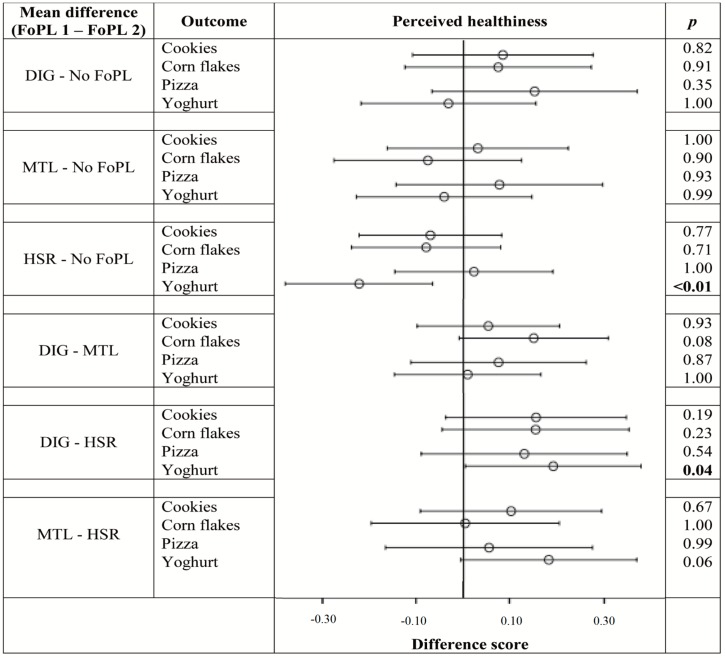
Difference in ratings between FoPLs (⊖) and the upper (├) and lower (┤) bounds of 95% confidence intervals for perceived healthiness according to food type.

**Figure 4:**
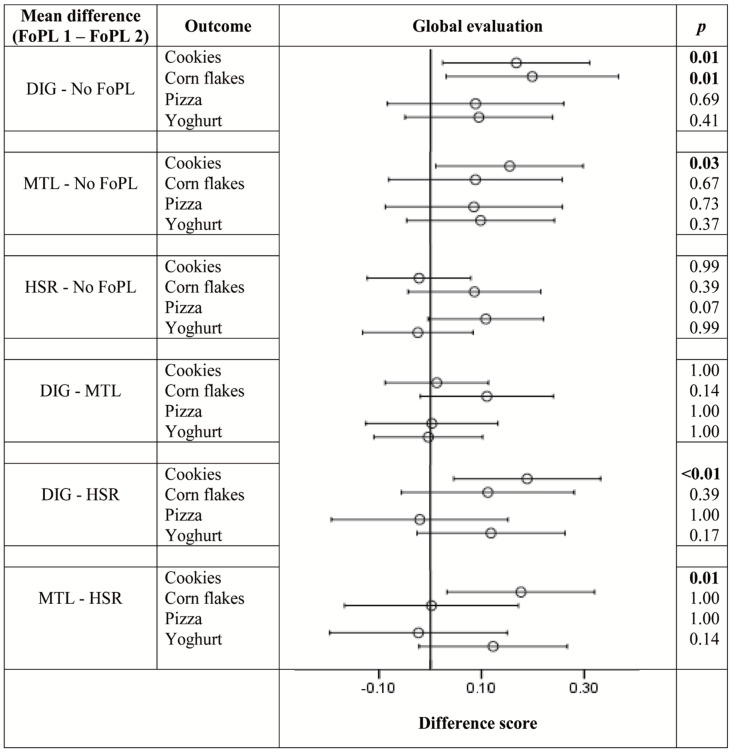
Difference in ratings between FoPLs (⊖) and the upper (├) and lower (┤) bounds of 95% confidence intervals for global evaluations according to food type.

**Figure 5:**
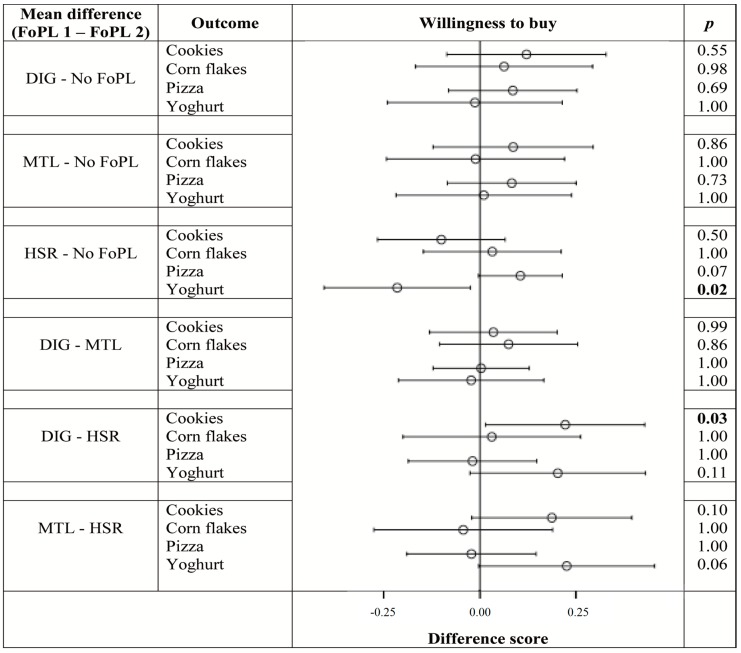
Difference in ratings between FoPLs (⊖) and the upper (├) and lower (┤) bounds of 95% confidence intervals for willingness to buy according to food type.

Figure 3, Figure 4 and Figure 5 illustrate the differences in outcomes between the FoPL conditions according to food type. The pattern of results observed within each of the food types did not always resemble the trends from the pooled data, indicating that the influence of FoPLs is likely to vary by product category.

Figure 3 shows that, in terms of perceived healthiness, there was no evidence of a positivity bias for any of the FoPLs within any individual food type. Yoghurts received the highest healthiness ratings of all the food types, particularly in the no FoPL condition. It was the only food type that differed according to FoPL, with the HSR leading to lower perceptions of healthiness than no FoPL.

Figure 4 shows that, in terms of global evaluations, the DIG produced a positivity bias for cookies and corn flakes, the MTL did so for cookies and the HSR produced no bias. The DIG and MTL both led to more positive evaluations than the HSR when applied to cookies. FoPLs did not have an effect on global evaluation ratings of pizzas and yoghurts.

Figure 5 shows that, in terms of willingness to buy, no positivity bias was observed for any of the food types. The HSR reduced willingness to buy yoghurts relative to no FoPL. Finally, the DIG led to greater perceptions of healthiness than the HSR in cookies.

A significant interaction was found between FoPLs and health claims across ratings of the yoghurt products. To further explore this interaction, the yoghurt data were split by health claim condition and linear mixed models were conducted with FoPL condition as the predictor variable (and age, gender and SES as covariates). Significant effects were followed up with planned comparisons. A significant effect (*p* = 0.038) was found in willingness to buy, with the HSR resulting in a lower willingness to buy than the DIG when a nutrient claim was present on pack.

## 6. Discussion

The present study sought to better understand the nature of the positivity bias, which could potentially lead consumers to judge unhealthy products with a health claim or FoPL more favourably than if this information was not present. Consumers’ responses to a range of unhealthy products showing varying combinations of health claims and FoPLs were assessed. Health claims were not a significant predictor of outcomes across the pooled data and only showed an effect (i.e., a positivity bias in response to higher level health claims) in willingness to buy cookies. Thus, hypothesis 1—that health claims would create a positivity bias in unhealthy foods—was not adequately supported. This does not necessarily indicate that health claims cannot produce a positivity bias, however they may be less persuasive in relation to unhealthy foods. This finding contributes to the limited and mixed literature on the effects of health claims on unhealthy foods [4,18,29,30,31,32,33].

By comparison, evidence was found that certain FoPLs (namely, the Daily Intake Guide and to a lesser extent the Multiple Traffic Light) are capable of creating a positivity bias by eliciting more favourable judgements of unhealthy foods compared to no FoPL. This finding held after demographic variables such as age, gender and SES were taken into account. Across the pooled data, and also specifically for the cookies, the Daily Intake Guide and Multiple Traffic Light led to more positive global evaluations compared to no FoPL. However, they did not differ from the no FoPL condition on perceived healthiness or willingness to buy. This is partly in line with previous studies on the Daily Intake Guide that have reported a positivity bias [2,31,47], but is at odds with studies on the Multiple Traffic Light that have reported reduced perceptions of healthiness when applied to unhealthy foods [31,43,44,45,46,48].

In the pooled data across all food types, the Health Star Rating did not result in judgements that were significantly different from the no FoPL condition, indicating that this FoPL did not cause a positivity bias. When examining specific food types, the Health Star Rating led to lower perceptions of healthiness than no FoPL in the case of yoghurts. This may be the result of consumers expecting yoghurts to be very healthy (as indicated by the no FoPL yoghurt products receiving the highest healthiness rating relative to the other food products). The Health Star Rating seems to have been the most effective FoPL in alerting participants to the poor nutritional profile of the yoghurt product shown, leading to lowered perceptions of healthiness, which then translated into lower willingness to buy. For yoghurts containing a nutrient content claim, the Health Star Rating led to a lower willingness to buy than the Daily Intake Guide. These results partly support those of Hamlin and McNeill [1], who also found that the Health Star Rating led to lower willingness to buy compared to no FoPL. However, in their study, this also applied to healthy products, which is likely to have been due to factors specific to that study, in particular the red colouring of the Health Star Rating.

The Daily Intake Guide (and the Multiple Traffic Light to a lesser extent) led to more favourable assessments of foods than the Health Star Rating. As one of the first quantitative studies to compare the Multiple Traffic Light to the Health Star Rating, these findings suggest that across multiple food types the Multiple Traffic Light is less likely to create a positivity bias than the Daily Intake Guide (in agreement with past research [31,44]), but is more likely to do so compared to the Health Star Rating.

Although the Multiple Traffic Light and Health Star Rating are both considered to be hybrid reductive/evaluative FoPLs, they resulted in different outcomes in the present study. Focus group research indicates that participants attend more to the HSR’s evaluative component (the star rating) than the reductive information provided (grams of nutrients per 100 grams) [37]. While the Multiple Traffic Light also contains evaluative (colours) and reductive (grams of nutrients) information, in the present study the Multiple Traffic Light generated results more like the reductive FoPL (i.e., the Daily Intake Guide). The overall summary indicator provided in the Health Star Rating compared to the nutrient-specific information provided in the Multiple Traffic Light and Daily Intake Guide may have facilitated more accurate product judgements and could thus explain the different findings between the three FoPLs. Given the mixed findings relating to hybrid FoPLs in this and the limited previous research, further work is needed to better understand how nutrient-specific, as opposed to summary-level, interpretive information affects the positivity bias.

Different types of food products (cookies, corn flakes, pizzas, yoghurt) were included in the present study to provide insight into possible differential effects by product category. The differences between FoPL conditions were much smaller (often non-significant) within each food type than across the pooled data. This was particularly the case for pizzas, where different types of FoPLs did not differentially affect participants’ judgments. The Daily Intake Guide and Multiple Traffic Light prompted significantly more favourable evaluations of the ostensibly unhealthier product (cookies), with little impact on the healthier product (yoghurt). These findings suggest that the product that was perceived to be less healthy received more of a promotional boost from these labelling schemes. In contrast, the Health Star Rating did not inflate appraisals of the unhealthy product. These results indicate that the presence and nature of the positivity bias varies across food types and that further research is needed to understand the factors determining why certain foods are more susceptible to this form of bias (e.g., pre-existing beliefs about healthiness).

Overall, these results provide partial support for the hypothesis that a reductive FoPL (Daily Intake Guide) can create a positivity bias compared to an evaluative FoPL (the Health Star Rating) and no FoPL. Importantly, this finding applied across a diverse sample of respondents. In this study, this positivity bias applied to global evaluations but not perceived healthiness or willingness to buy.

### 6.1. Limitations

A limitation of this study is that participants evaluated one product at a time. As such, the insights gained are more relevant to non-comparative processing contexts where products are evaluated individually rather than simultaneously compared with other products. Research indicates that processing strategies vary between comparative and non-comparative contexts [67]. For example, a stronger positivity bias towards health claims may occur in scenarios where consumers have to choose whether to purchase a product with or without a health claim from within a choice set as opposed to evaluating each product holistically. Given that the comparative context more closely resembles the real world, further research involving the simultaneous assessment of multiple products would be informative. Another limitation of the design of this study was the use of a web panel. This scenario cannot replicate a real shopping environment in which consumers are fully engaged in the decision making process and also subject to distractions and time pressures. Thus, further behavioural research is required. Assessing only four product types may limit the generalizability of the insights from this current study. However, the products studied represent a diverse range of core, discretionary, meal and snack foods.

### 6.2. Implications

The findings of the present study suggest that health claims have less impact on food product evaluations of unhealthy foods than FoPLs. Furthermore, there was little evidence that health claims created a positivity bias in the current sample. Conversely, the presence of the Daily Intake Guide and, to a lesser extent, the Multiple Traffic Light can result in consumers arriving at more favourable judgments of unhealthy foods compared to when no FoPL or a Health Star Rating is present. Of all the outcome variables assessed, FoPLs had greatest impact on global evaluations and a comparatively weaker effect on perceived healthiness and willingness to buy. This suggests that the positivity bias is borne out more in generalized impressions of a product and less so in people’s behavioural intentions. Nevertheless, this bias is of concern for the public health sector as it indicates that certain forms of nutrition information on food packs may lead to elevated positive judgments of unhealthy foods. If consumers are unable or unmotivated to deliberatively interpret the nutritional information contained within a FoPL, it is likely that their product assessments will be more strongly influenced by the presence of a FoPL rather than the nutrition information per se.

The positivity bias for unhealthy foods created by the Multiple Traffic Light was unexpected given that numerous reviews have concluded it is one of the more effective FoPLs in terms of helping participants identify and select healthier foods [36,40,41,68,69]. The conclusions of these reviews are not necessarily negated by the present findings because of the specific nature of the study (i.e., the inclusion of only unhealthy foods and the methodological approach of requiring respondents to assess one product at a time). It is possible that the Multiple Traffic Light allows consumers to differentiate between relative healthiness when viewing multiple products simultaneously but is less effective at facilitating identification of unhealthy products when they are viewed in isolation.

The Health Star Rating was the only FoPL that did not lead to a positivity bias for unhealthy food products, which may have been due to the ease of interpreting the information provided in its summary indicator [59]. Given that attention to and understanding of FoPLs is known to depend on familiarity [39,45], this finding is unexpected as respondents in the present study would have been least familiar with this FoPL. This suggests that ease of use may be more important to understanding than familiarity. These findings support the Australian government’s decision to adopt the Health Star Rating.

Although the size of the differences detected in this study may be considered small (e.g., a difference of 0.1 on a 5 point scale), this is to be expected given that factors such as taste, price and convenience often have a greater impact on product evaluations than healthiness [70], and previous studies also generally report small changes [48,71,72]. In these experimental studies, exposure to the labelling intervention is relatively brief. It is possible that with repeated exposure to such claims in the marketplace, the impact would be more marked. However, further research with varying “doses” of exposure would be required to determine whether impacts of particular food labelling schemes increase with repeated exposure or decay after an initial impact.

## 7. Conclusions

To conclude, the results of the present study indicate that reductive FoPLs can potentially lead to more positive evaluations of unhealthy products compared to no FoPL. Further behavioural research is needed to determine the extent to which this may lead to unhealthy foods being consumed at a higher rate than they otherwise would be or at a higher rate compared to healthier products without a FoPL.

## Figures and Tables

**Table 1:** Number of participants within each demographic category (*n* = 1984).

Males (*n* = 988)			Females (*n* = 996)		
Age (Years)	Socio-Economic Status *		Age (Years)	Socio-Economic Status *	
	Low (*n* = 483)	Medium-High (*n* = 505)		Low (*n* = 497)	Medium-High (*n* = 499)
10–14	63	60	10–14	63	60
15–18	65	71	15–18	59	68
19–25	52	53	19–25	55	54
26–35	62	64	26–35	62	65
36–45	60	64	36–45	64	64
46–55	56	61	46–55	66	59
56–65	61	67	56–65	63	65
65+	64	65	65+	65	64

* Low Socio-Economic Status category comprised those in Socio-Economic Indexes for Areas (SEIFA) deciles 1 to 4 [63].

**Table 2:** Means, main effects and interaction effects for front-of-pack labels and health claims (with age, gender and Socio-Economic Status as covariates) on perceived healthiness, global evaluations and willingness to buy across all foods (*n* = 1984).

	Perceived Healthiness	Global Evaluation	Willingness to Buy
Front-of-pack label	*F*(3, 4446.3) = 5.3, *p* < 0.001	*F*(3, 4212.6) = 12.4, *p* < 0.001	*F*(3, 4264.1) = 4.3, *p* < 0.001
None	2.94	3.41	2.91
DIG	3.02	3.54	2.99
MTL	2.96	3.53	2.97
HSR	2.88	3.46	2.86
Health claims	*F*(3, 4759.43) = 0.2, *p* = 0.86	*F*(3, 4464.8) = 1.4, *p* = 0.25	*F*(3, 4534.8) = 2.3, *p* = 0.08
None	2.94	3.48	2.88
Nutrient content	2.94	3.47	2.92
General level health claim	2.96	3.52	2.97
Higher level health claim	2.97	3.48	2.96
Front-of-pack label × health claim	*F*(9, 4811.6) = 0.6, *p* = 0.80	*F*(9, 4508.7) = 0.5, *p* = 0.85	*F*(9, 4581.5) = 0.2, *p* = 0.99

DIG: Daily Intake Guide; MTL: Multiple Traffic Lights; HSR: Health Star Rating.

**Table 3:** Means, main effects and interaction effects for front-of-pack labels and health claims (with age, gender and Socio-Economic Status as covariates) on perceived healthiness, global evaluations and willingness to buy according to food type (*n* = 1984).

	Perceived Healthiness	Global Evaluation	Willingness to Buy
**Cookies**			
Front-of-pack label	*F*(3, 745.2) = 1.6, *p* = 0.194	*F*(3, 586.8) = 4.5, *p* < 0.001	*F*(3, 741.3) = 2.8, *p* = 0.038
None	2.59	3.54	3.00
DIG	2.67	3.70	3.12
MTL	2.62	3.69	3.08
HSR	2.52	3.52	2.90
Health claims	*F*(3, 842.1) = 1.2, *p* = 0.308	*F*(3, 617.2) = 0.6, *p* = 0.607	*F*(3, 843.1) = 3.3, *p* = 0.021
None	2.55	3.61	2.93
Nutrient content	2.57	3.60	3.06
General level health claim	2.58	3.59	2.97
Higher level health claim	2.67	3.65	3.14
Front-of-pack label × health claim	*F*(9, 824.7) = 1.6, *p* = 0.120	*F*(9, 603.9) = 0.5, *p* = 0.870	*F*(9, 825.4) = 0.5, *p* = 0.863
**Corn flakes**			
Front-of-pack label	*F*(3, 736.3) = 3, *p* = 0.031	*F*(3, 679.5) = 3.8, *p* = 0.010	*F*(3, 696.5) = 0.5, *p* = 0.683
None	3.22	3.28	2.96
DIG	3.29	3.47	3.03
MTL	3.14	3.36	2.95
HSR	3.13	3.35	2.99
Health claims	*F*(3, 800.2) = 0.6, *p* = 0.609	*F*(3, 722.5) = 0.4, *p* = 0.775	*F*(3, 748) = 0.8, *p* = 0.483
None	3.15	3.33	2.92
Nutrient content	3.18	3.37	2.96
General level health claim	3.24	3.39	3.03
Higher level health claim	3.21	3.36	3.01
Front-of-pack label × health claim	*F*(9, 803.5) = 1.2, *p* = 0.269	*F*(9, 725.7) = 0.9, *p* = 0.516	*F*(9, 751.2) = 0.6, *p* = 0.822
**Pizza**			
Front-of-pack label	*F*(3, 623.3) = 1.2, *p* = 0.304	*F*(3, 449.5) = 2.5, *p* = 0.06	*F*(3, 588.6) = 0.5, *p* = 0.69
None	2.54	3.41	3.01
DIG	2.69	3.50	3.00
MTL	2.62	3.50	2.98
HSR	2.57	3.52	2.93
Health claims	*F*(3, 663.6) = 0.2, *p* = 0.892	*F*(3, 458.2) = 1.4, *p* = 0.229	*F*(3, 620.4) = 1, *p* = 0.377
None	2.58	3.45	2.93
Nutrient content	2.63	3.47	2.95
General level health claim	2.62	3.55	3.07
Higher level health claim	2.58	3.45	2.95
Front-of-pack label x health claim	*F*(9, 677.6) = 1.5, *p* = 0.16	*F*(9, 464.8) = 1, *p* = 0.43	*F*(9, 632.3) = 1, *p* = 0.433
**Yoghurt**			
Front-of-pack label	*F*(3, 817.9) = 4.9, *p* = 0.002	*F*(3, 615.6) = 1.9, *p* = 0.129	*F*(3, 791.9) = 4, *p* = 0.008
None	3.60	3.61	3.14
DIG	3.58	3.70	3.12
MTL	3.57	3.72	3.15
HSR	3.39	3.60	2.92
Health claims	*F*(3, 955.5) = 0.8, *p* = 0.505	*F*(3, 669.9) = 0.3, *p* = 0.817	*F*(3, 930.6) = 0.1, *p* = 0.973
None	3.60	3.64	3.14
Nutrient content	3.57	3.68	3.12
General level health claim	3.57	3.66	3.15
Higher level health claim	3.39	3.64	2.92
Front-of-pack label × health claim	*F*(9, 921.6) = 1.9, *p* = 0.048	*F*(9, 639.8) = 2.5, *p* = 0.009	*F*(9, 895.7) = 1.9, *p* = 0.048

DIG: Daily Intake Guide; MTL: Multiple Traffic Lights; HSR: Health Star Rating.
